# Association between the non-high-density lipoprotein cholesterol to high-density lipoprotein cholesterol ratio and cognitive impairment in patients with cerebral small vessel disease

**DOI:** 10.3389/fneur.2025.1703244

**Published:** 2026-01-05

**Authors:** Chao Wang, Zhenjie Teng, Mingyue Fan, Xiaohua Xie, Weihong Chen, Yueshan Zhao, Jiayu Zhang, Yanhong Dong, Jing Xu, Wei Jin, Peiyuan Lv

**Affiliations:** 1 Department of Neurology, Graduate School of North China University of Science and Technology, Tangshan, China; 2 Department of Neurology, Hebei General Hospital, Shijiazhuang, China; 3 Hebei Provincial Key Laboratory of Cerebral Networks and Cognitive Disorders, Shijiazhuang, China

**Keywords:** cerebral small vessel disease, cognitive impairment, high-density lipoprotein cholesterol, non-high-density lipoprotein cholesterol, non-high-density lipoprotein cholesterol to HDL-C ratio

## Abstract

**Objective:**

This study aims to determine the potential association between non-high-density lipoprotein cholesterol to high-density lipoprotein cholesterol ratio (NHHR) and cognitive impairment in patients with cerebral small vessel disease (CSVD). By collecting data from patients with CSVD in hospital, we will analyze the relationship between non-high-density lipoprotein cholesterol to high-density lipoprotein cholesterol ratio and cognitive function in these patients.

**Methods:**

This study enrolled 263 CSVD patients, Cognitive function was assessed using Mini-Mental State Examination (MMSE) within 2 weeks, with cognitive impairment defined by education stratified thresholds. Statistical analysis of the baseline was performed. The association between NHHR and cognitive function was evaluated using binary logistic regression. And Receiver Operating Characteristic Curve (ROC) analysis were performed to evaluate the predictive value.

**Results:**

Patients were classified into cognitive impairment group (*n* = 138) and normal cognition group (*n* = 125). NHHR in the cognitive impairment group was significantly higher than that in the normal group (3.58 ± 0.98 vs. 2.90 ± 0.92, *P* < 0.001). There was a dose–response relationship between NHHR quartiles and the incidence of cognitive impairment (trend test *P* < 0.001). Multivariate regression analysis showed that for each unit increase in NHHR, the risk of cognitive impairment increases by 184% (OR = 2.84, 95% confidence interval 1.97 to 4.12; *p* < 0.001). The predictive model constructed by combining age and education level has an area under the ROC curve (AUC) of 0.703.

**Conclusion:**

NHHR serves as an independent predictor of cognitive impairment in CSVD patients. The NHHR-derived model exhibits moderate discriminative accuracy, indicating potential clinical applicability.

## Introduction

With increasing life expectancy, age-related cognitive decline may pose a significant health challenge for the elderly population (1). For elderly patients presenting with cognitive impairment, when other disorders associated with cognitive impairment have been excluded, cerebral small vessel disease (CSVD) should be considered by clinicians as an important potential factor (2). CSVD refers to a clinical, imaging, and pathological syndrome caused by various etiologies affecting the small arteries, arterioles, capillaries, venules, and small veins within the brain. It is clearly recognized as one of the primary causes of vascular cognitive impairment (3). CSVD serves as the major pathological substrate for vascular-mediated cognitive decline and dementia (4), and it affects approximately 60% of individuals aged 65 and older (5). Consequently, early screening for CSVD-related cognitive impairment and identifying its risk factors are of paramount importance.

Non-high-density lipoprotein cholesterol to high-density lipoprotein cholesterol ratio (NHHR) is a newly developed atherogenic lipid composite index (6) that demonstrates superior performance compared to traditional lipid parameters in assessing the risk of cardiovascular and cerebrovascular diseases. Dyslipidemia affects cognitive function in ischemic stroke patients by accelerating the progression of systemic atherosclerosis and is recognized as a significant risk factor for cognitive impairment and even dementia (7, 8). Previous studies have indicated that NHHR exhibits enhanced predictive and diagnostic capabilities over conventional lipid parameters for assessing the risk of conditions such as atherosclerosis, non-alcoholic fatty liver disease, chronic kidney disease, diabetes mellitus, depression, and metabolic syndrome (9–12). Recently, research has demonstrated the moderate predictive ability of NHHR for cognitive impairment in patients with acute ischemic stroke (13). However, no studies have yet investigated the predictive role of NHHR for cognitive impairment specifically in CSVD patients.

In this study, we explored the association between NHHR and cognitive impairment in patients with CSVD, aiming to provide valuable insights for the early prevention and monitoring of cognitive impairment in this population.

## Methods

### Study population

This cross-sectional investigation retrospectively analyzed data from inpatients at Hebei Provincial People’s Hospital between April 2024 and May 2025. We enrolled patients aged ≥40 years who underwent blood tests, completed cognitive assessments, and received brain MRI to assess neuroimaging markers of CSVD. All patients were administered the same lipid-lowering therapy. Exclusion criteria comprised: (1) Active infection within the preceding 2 weeks or current antibiotic use; (2) Diagnosed hematological diseases, malignancies, or autoimmune disorders; (3) Recent immunosuppressive therapy; (4) Comorbid systemic disorders potentially causing cognitive impairment (e.g., thyroid dysfunction, severe anemia, hepatic/renal insufficiency, malignancy, alcohol/substance abuse); (5) Major psychiatric disorders including schizophrenia or severe anxiety/depression; (6) Inability to complete cognitive evaluations. Ultimately, 263 eligible participants were included in the analysis. The study protocol conformed to the Declaration of Helsinki and received approval from the Hebei General Hospital Research Ethics Committee (Approval No. 2025-LW-0192).

### Clinical characteristics

All demographic characteristics and risk factors were obtained from medical records: age, sex, education years, body mass index (BMI), smoking status, and alcohol consumption. Medical history, including hypertension, diabetes mellitus, coronary heart disease (CHD), and stroke, was also collected. Laboratory biomarkers were assayed (blood samples were obtained within 24 h of hospital admission): Fasting blood glucose (FBG), Total cholesterol (TC), High-density lipoprotein cholesterol (HDL-C), Low-density lipoprotein cholesterol (LDL-C), Uric acid (UA), White blood cell count (WBC), Neutrophil count, Lymphocyte count, Platelet count. The Systemic Immune-Inflammation Index (SII) was calculated as: SII = (Platelet count × Neutrophil count)/Lymphocyte count (14). Non-high-density lipoprotein cholesterol (Non-HDL-C) was calculated as: Non-HDL-C = TC – HDL-C. The NHHR was calculated as: NHHR = Non-HDL-C/HDL-C (6).

### Imaging acquisition and assessment

All participants underwent brain MRI using 3.0-Tesla scanners (Signa, GE Healthcare, United States). The standardized imaging protocol included the following sequences with specific parameters: T1-weighted imaging (T1WI): TR/TE 1909/20.2 ms, FOV 240 × 192 mm^2^, matrix 320 × 224, NEX 1; T2-weighted imaging (T2WI): TR/TE 5000/125 ms, FOV 240 × 240 mm^2^, matrix 352 × 352, NEX 1; Fluid-attenuated inversion recovery (FLAIR): TR/TE 8502/159.4 ms, FOV 240 × 240 mm^2^, matrix 256 × 256, NEX 1; Susceptibility weighted imaging (SWI): TR/TE 78.6/47.6 ms, FOV 240 × 216 mm^2^, matrix 384 × 320, NEX 1 (Slice thickness: 2 mm); Diffusion-weighted imaging (DWI): TR/TE 4800/81.7 ms, FOV 240 × 240 mm^2^, matrix 160 × 160, NEX 1. Slice thickness was 5 mm for T1WI, T2WI, FLAIR, and DWI sequences.

Imaging evaluations were independently performed by two neurologists who were blinded to all other clinical data. Both assessors possessed over 5 years of specialized experience in neuroimaging diagnosis of cognitive impairment disorders. In cases of discrepant interpretations, a senior neuroradiology expert with more than 20 years of experience served as the final arbiter. The total CSVD burden score ranged from 0 to 4 points. One point was assigned for the presence of each of the following features (15): (1) ≥ 1 lacune; (2) Periventricular white matter hyperintensity (WMH) graded as Fazekas score 3 and/or deep WMH graded as Fazekas score ≥2; (3) ≥ 1 cerebral microbleed; (4) Moderate-to-severe (grade 2–4) perivascular spaces (PVS) in the basal ganglia. Based on the total CSVD burden score, patients were stratified into two groups: MRI low-burden group: scores 0–1, MRI high-burden group: scores 2–4.

### Neuropsychological assessment

Neuropsychological assessment using the validated Chinese Mini-Mental State Examination (MMSE) was completed for all participants, with results sourced from medical records. Neuropsychological evaluations were conducted by a neuropsychologist who had received systematic training. To minimize potential bias, the examiner remained blinded to all subject clinical and radiological information. Given the strong influence of education on MMSE performance, cognitive impairment diagnosis incorporated education-stratified cut-offs based on Chinese population norms: ≤17 points: Uneducated individuals; ≤20 points: 1–6 years of education; ≤24 points: >7 years of education (16).

### Statistical analyses

Statistical analyses were performed using SPSS 26.0 (IBM, Armonk, NY, United States). Continuous variables are presented as mean ± standard deviation for normally distributed data, analyzed with the two-tailed Student’s *t*-test, or as median (interquartile range) for non-normally distributed data, analyzed using the Mann–Whitney *U* test. Categorical variables are expressed as frequencies (percentages) and compared between groups with the *χ*^2^ test. The association between NHHR and cognitive function was evaluated using binary logistic regression. The predictive performance of NHHR levels for cognitive impairment was quantified by generating ROC curves.

## Results

### Participant characteristics

A total of 263 patients (median age: 62 years, interquartile range: 54–70 years; 178 males and 85 females) were included in the current analysis. Based on MMSE scores and years of education, the cognitive impairment group comprised 138 patients, while the cognitively normal group comprised 125 patients. Detailed information on these two groups is provided in Table 1.

**Table 1:** Baseline clinical characteristics of the study population stratified by cognitive status.

Clinical characteristics	Cognitive impairment group	NO cognitive impairment	*p*-value
	(*n* = 138)	(*n* = 125)	
Sex, female, *n* (%)	48 (34.8)	37 (29.6)	0.429
BMI, median (IQR), kg/m^2^	25.5 (22.6–28.1)	25.8 (23.7–27.7)	0.567
Age, median (IQR), years	66.0 (59–71.3)	59.0 (51–66.5)	<0.001*
Hypertension, *n* (%)	95 (68.8)	80 (64.0)	0.434
Diabetes, *n* (%)	40 (29.0)	38 (30.4)	0.893
Coronary heart disease, *n* (%)	15 (10.9)	11 (8.8)	0.68
History of stroke, *n* (%)	31 (22.5)	39 (31.2)	0.125
History of cerebral hemorrhage, *n* (%)	6 (4.3)	5 (4.0)	1
Hyperlipidemia, *n* (%)	34 (24.6)	17 (13.6)	0.029*
Smoking History, *n* (%)	40 (29.0)	41 (32.8)	0.507
Alcohol Use History, *n* (%)	23 (16.7)	31 (24.8)	0.126
MRI severe burden group, *n* (%)	100 (72.5)	60 (48.0)	0.001*
WBC Count, median (IQR), 10^9^/L	6.7 (5.7–8.5)	6.2 (5.5–7.7)	0.044*
Neutrophil Count, median (IQR), 10^9^/L	4.6 (3.6–6.3)	4.1 (3.3–5.6)	0.044*
Lymphocyte Count, median (IQR), 10^9^/L	1.6 (1.3–2.0)	1.7 (1.3–2.1)	0.687
FBG, median (IQR), mmol/L	6.1 (5.4–8.0)	5.5 (4.7–6.7)	<0.001*
SII, median (IQR), 10^9^/L	721.0 (595.5–857.3)	598.0 (448–780.5)	<0.001*
TC, median (IQR), mmol/L	4.8 (4.0–5.8)	4.6 (3.8–5.4)	0.057
HDL-C, median (IQR), mmol/L	1.1 (0.9–1.3)	1.1 (1.0–1.4)	0.006*
LDL-C, median (IQR), mmol/L	2.8 (2.2–3.5)	2.6 (2.0–3.2)	0.031*
LDL-C/HDL-C, median (IQR)	2.7 (2.2–3.1)	2.2 (1.7–2.7)	<0.001*
Hemoglobin, mean (SD), g/L	136.25 ± 17.04	137.24 ± 17.09	0.64
Platelet Count, mean (SD), 10^9^/L	252.64 ± 59.49	229.67 ± 56.82	0.002*
Uric Acid, mean (SD), μmol/L	306.91 ± 92.57	340.03 ± 106.41	0.007*
TC/HDL-C, mean (SD)	4.58 ± 0.98	3.90 ± 0.92	<0.001*
Non-HDL-C, mean (SD), mmol/L	3.90 ± 1.21	3.45 ± 1.10	0.002*
NHHR, mean (SD)	3.58 ± 0.98	2.90 ± 0.92	<0.001*

*p < 0.05.

SD, standard deviation; IQR, interquartile range; BMI, body mass index; WBC, White Blood Cell Count; TC, total cholesterol; HDL-C, high density lipoprotein cholesterol; LDL-C, low density lipoprotein cholesterol; SII, systemic immune-inflammation index; FBG, fasting blood glucose.

Compared with patients without cognitive impairment, those with cognitive impairment were significantly older (*p* < 0.001). Statistically significant differences (*p* < 0.05) were observed between the two groups in Hyperlipidemia, MRI severe burden group, White blood cell count, Neutrophil count, Fasting blood glucose, SII, HDL-C, LDL-C, Non-HDL-C, LDL-C/HDL-C, Platelet count, Uric acid, TC/HDL-C, NHHR. The remaining demographic and general clinical data showed no statistically significant differences between the two groups.

### The relationship between NHHR and cognitive impairment

**Figure 1:**
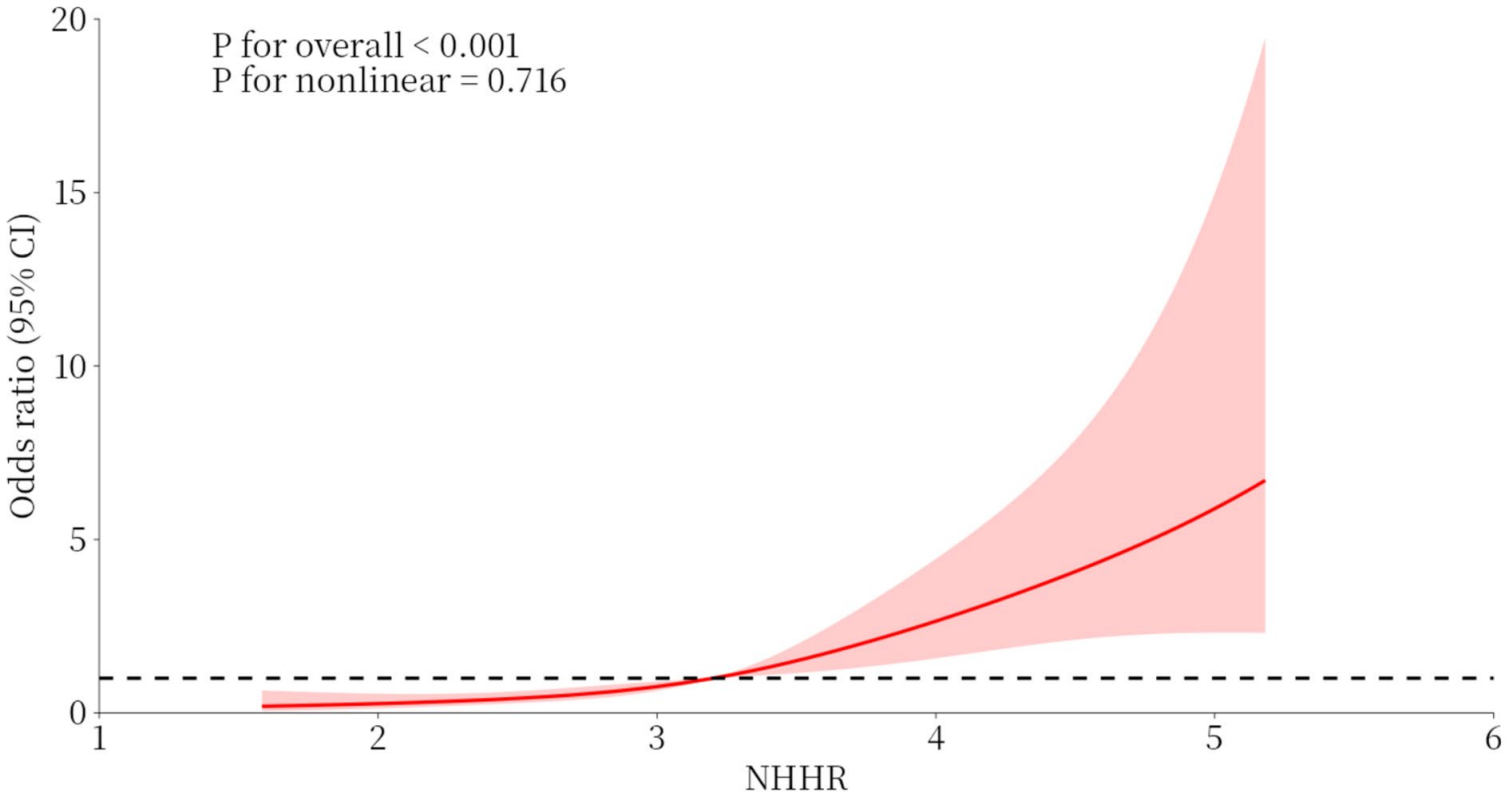
Restricted cubic spline for the association between NHHR and the risk of cognitive impairment.

In this study, we used a logistic regression model to explore the relationship between NHHR and cognitive impairment (Table 2). After adjusting for age, SII, hyperlipidemia, UA, MRI severe burden group and FBC, the logistic regression results showed that NHHR was significantly associated with the occurrence of cognitive impairment. Each unit increase in NHHR was independently associated with a 184% elevated risk of cognitive impairment (OR: 2.84; 95% CI: 1.97 to 4.12; *p* < 0.001). By comparison, although increased age (OR = 1.08, 95% CI: 1.04–1.11, *p* < 0.001) and elevated SII (OR = 1.01, 95% CI: 1.00–1.01, *p* = 0.011) were also statistically significant, their effect sizes were substantially smaller than that of NHHR (representing approximately 8 and 1% increased risk per unit increase, respectively). Furthermore, hyperlipidemia (OR = 2.71, 95% CI: 1.18–6.20, *p* = 0.018) and uric acid levels (OR = 0.99, 95% CI: 0.99–0.99, *p* = 0.002) were identified as protective factors. Critically, the robust association of NHHR remained independent of non-significant factors, including severe white matter hyperintensity burden on MRI (OR = 1.21, 95% CI: 0.56–2.60, *p* = 0.634) and fasting blood glucose levels (OR = 1.057, 95% CI: 0.94–1.19, *p* = 0.362) Additionally, the results of the restricted cubic spline graph more clearly demonstrate the dose–response curve of NHHR and the risk of cognitive impairment (Figure 1). After adjusting for all confounding factors, a significant linear dose–response relationship was observed between NHHR and cognitive impairment (*P* for overall association < 0.001; *P* for nonlinearity = 0.716). Cognitive impairment risk increased progressively with higher NHHR, reaching an ~6-fold higher odds at NHHR = 5 versus the reference (NHHR = 1) (Figure 1).

**Table 2:** Multivariable logistic regression analysis of factors associated with cognitive impairment.

Factors	Multivariable analysis	
	OR (95% CI)	*P*-value
Age	1.08 (1.04 ~ 1.11)	<0.001*
SII	1.01 (1.00 ~ 1.01)	0.011*
Hyperlipidemia	2.71 (1.18 ~ 6.20)	0.018*
Uric-acid	0.99 (0.99 ~ 0.99)	0.002*
NHHR	2.84 (1.97 ~ 4.12)	<0.001*
MRI severe burden group	1.21 (0.56 ~ 2.60)	0.634
Fasting blood glucose	1.057 (0.94 ~ 1.19)	0.362

*P < 0.05.

**Figure 2:**
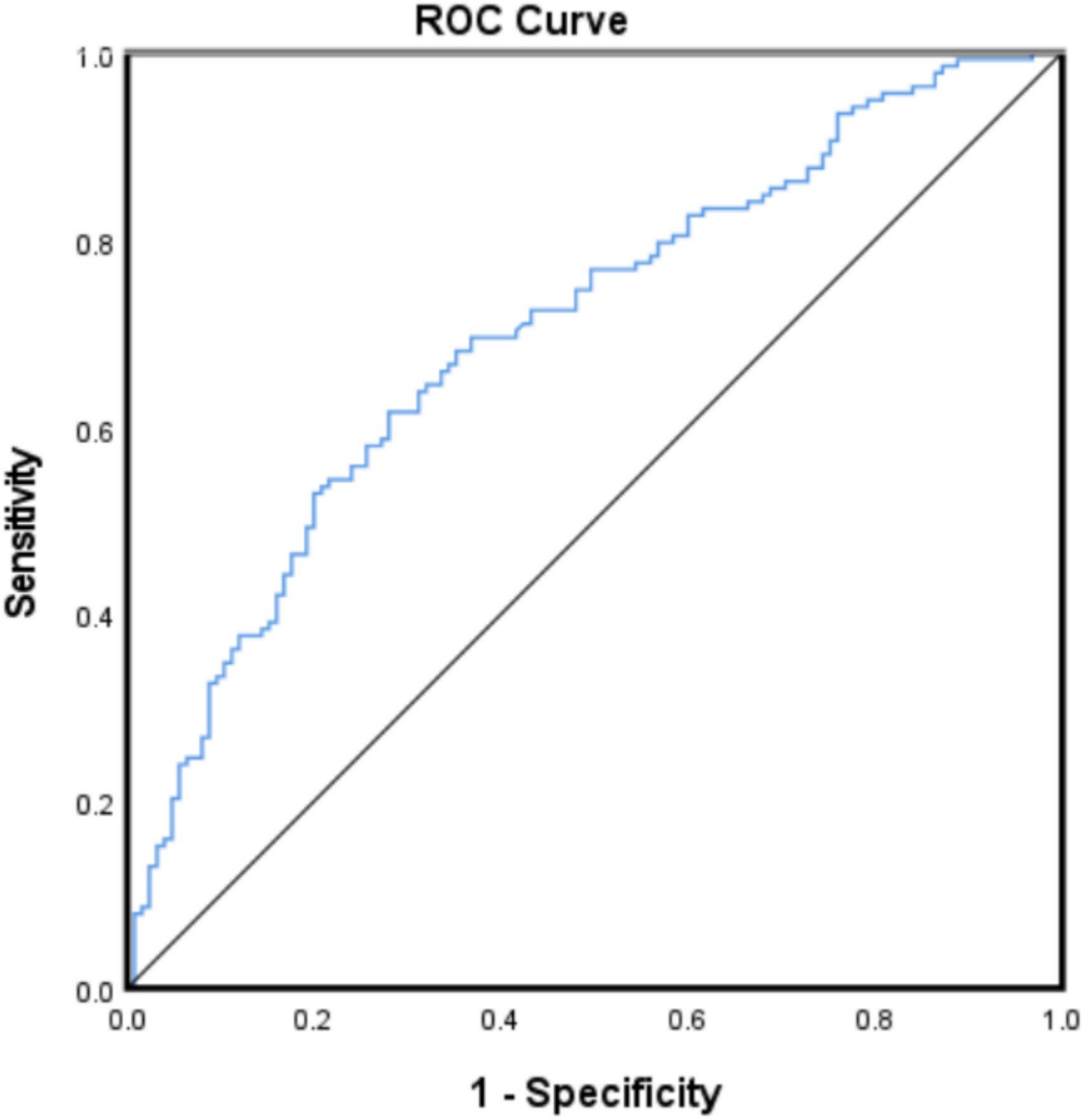
Receiver operating characteristic (ROC) curve of NHHR levels for cognitive impairment.

The ROC curve analysis demonstrated NHHR’s significant predictive capability for cognitive impairment (AUC = 0.703; 95% CI: 0.640–0.765, *p* < 0.001). At the optimal cutoff of 3.312, NHHR achieved balanced sensitivity and specificity, confirming its utility as a clinically relevant risk indicator (Figure 2).

In the unadjusted model (Model 1): Compared with NHHR grade 1 (reference group), grade 2 showed no significant difference in the risk of cognitive impairment (OR = 1.69, 95% CI: 0.92–3.09, *p* = 0.089). The risk for grade 3 increased significantly by 5.03-fold (OR = 5.03, 95% CI: 2.63–9.61, *p* < 0.001). Trend test: For each one-grade increase in NHHR, the risk increased by 2.22-fold (OR = 2.22, 95% CI: 1.64–3.00, *p* < 0.001).

After adjusting for age and SII (Model 2): The risk for grade 2 remained non-significant (OR = 1.90, 95% CI: 0.98–3.68, *p* = 0.059). The risk for grade 3 further increased to 6.46-fold (OR = 6.46, 95% CI: 3.11–13.42, *p* < 0.001). Trend test: For each one-grade increase in NHHR, the risk increased by 2.63-fold (OR = 2.63, 95% CI: 1.86–3.73, *p* < 0.001).

After further adjustment for uric acid (Model 3): The risk for grade 2 became significantly elevated (OR = 2.03, 95% CI: 1.03–4.00, *p* = 0.040). The risk for grade 3 jumped to 8.01-fold (OR = 8.01, 95% CI: 3.72–17.24, *p* < 0.001). Trend test: For each one-grade increase in NHHR, the risk increased by 2.95-fold (OR = 2.95, 95% CI: 2.05–4.24, *p* < 0.001) (Table 3).

**Table 3:** Multivariable logistic regression analysis of the association between NHHR tertiles and cognitive impairment.

Variables	Model 1		Model 2		Model 3	
	OR (95%CI)	*P*	OR (95%CI)	*P*	OR (95%CI)	*P*
NHHR three						
1	1.00 (Reference)		1.00 (Reference)		1.00 (Reference)	
2	1.69 (0.92 ~ 3.09)	0.089	1.90 (0.98 ~ 3.68)	0.059	2.03 (1.03 ~ 4.00)	**0.040**
3	5.03 (2.63 ~ 9.61)	**<0.001**	6.46 (3.11 ~ 13.42)	**<0.001**	8.01 (3.72 ~ 17.24)	**<0.001**
*P* for trend	2.22 (1.64 ~ 3.00)	**<0.001**	2.63 (1.86 ~ 3.73)	**<0.001**	2.95 (2.05 ~ 4.24)	**<0.001**

OR, odds ratio; CI, confidence interval; Model 1: Crude; Model 2: Adjust: age, SII; Model 3: Adjust: age, SII, Uric-acid. The bold values represent the *P*-values that indicate a statistically significant association (*P* < 0.05) between NHHR tertiles and cognitive impairment in the corresponding model.

**Figure 3:**
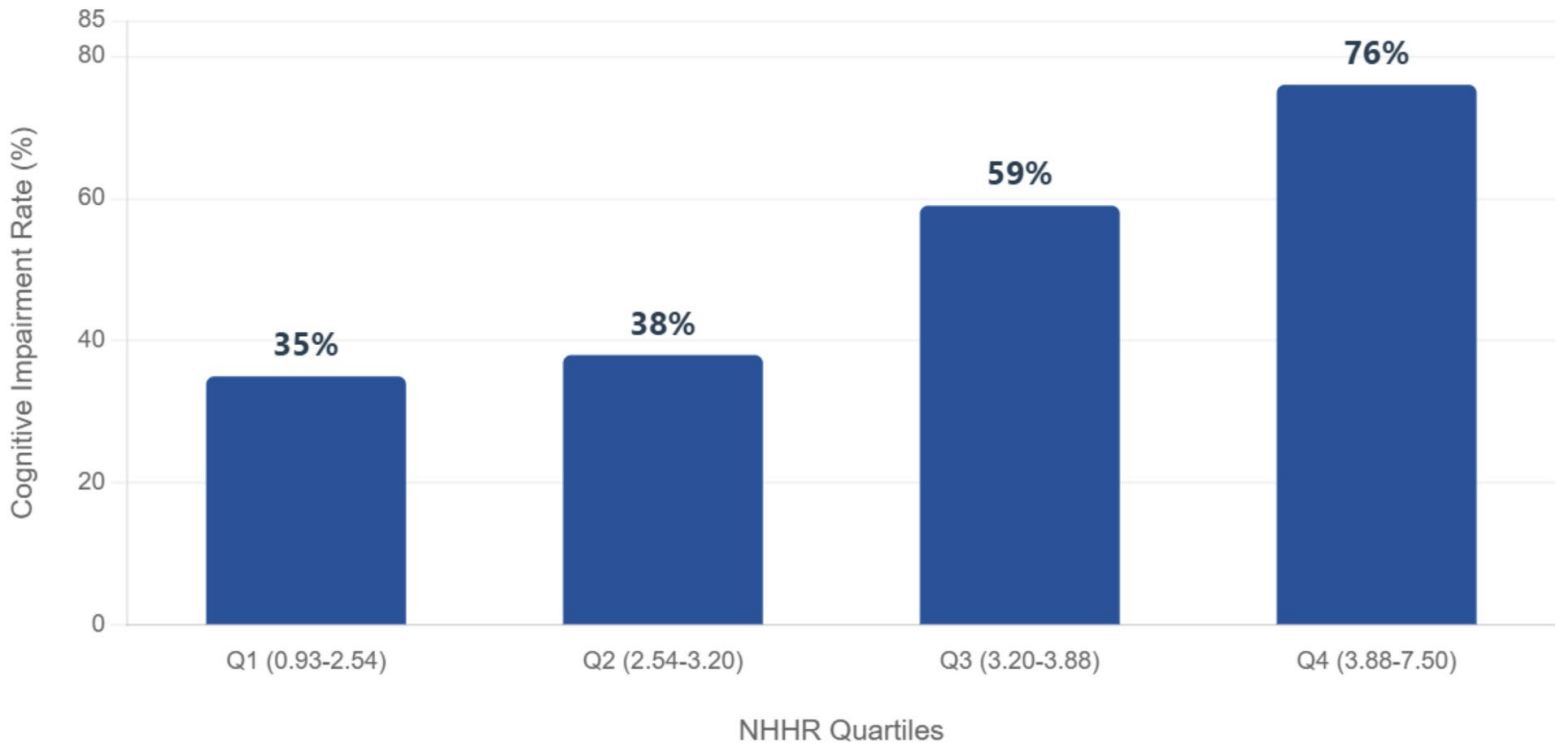
Prevalence of cognitive impairment according to NHHR quartiles.

A strong dose-dependent relationship was observed between increasing NHHR quartiles and cognitive impairment prevalence. Participants in the highest NHHR quartile (Q4: 3.88–7.50, 95% CI: 0.75–0.93) exhibited a substantially elevated cognitive impairment rate of 76%, which was more than double the rate in the lowest quartile (Q1: 0.93–2.54, 35, 95% CI: 0.47–0.71). Rates increased progressively across quartiles: Q2 (2.54–3.20, 95% CI: 0.56–0.79) showed a 38% prevalence, while Q3 (3.20–3.88, 95% CI: 0.64–0.85) demonstrated a pronounced rise to 59%. This trend indicates that higher NHHR levels are robustly associated with greater cognitive impairment risk (Figure 3).

## Discussion

This study identified NHHR as an independent risk factor for cognitive impairment with a robust dose–response relationship. After full adjustment (Model 3), the highest NHHR grade (Grade 3) was associated with an 8.01-fold increased risk of cognitive impairment (95% CI: 3.72–17.24), and the risk escalated significantly per grade increment (trend-test OR = 2.95 per grade, *p* < 0.001). ROC analysis confirmed NHHR’s predictive capacity (AUC = 0.703), with an optimal cutoff of 3.312 supporting clinical risk stratification. It is noteworthy that across NHHR quartiles, the risk of cognitive impairment ranged from 35 to 76%, indicating that elevated NHHR levels correlate with a higher likelihood of cognitive dysfunction. Our predictive model (AUC = 0.703) enables rapid cognitive risk screening upon admission using routine lipid profiles. High-risk patients may benefit from early cognitive training and intensified lipid-lowering therapy interventions.

To the best of our knowledge, currently, no studies have investigated the relationship between NHHR and cognitive function in patients with CSVD. Recent evidence demonstrates that each 1-unit increase in NHHR is associated with a 13.2% elevated risk of cognitive impairment in patients with acute ischemic stroke (13). Furthermore, Ma et al. (17) established a positive association between NHHR and stroke prevalence, suggesting its potential as a novel predictive biomarker for stroke.

The impact of NHHR on cognitive function may involve the following mechanisms. Lipoproteins play a critical role in atherosclerosis (18). Non-HDL-C can induce reactive oxygen species (ROS) generation and activate the NF-κB/NLRP3 pathway, triggering oxidative stress and promoting *β*-amyloid (Aβ) deposition (19). Non-HDL-C demonstrates a stronger correlation with cardiovascular risk than LDL-C alone (20). Conversely, HDL-C exerts protective effects against atherosclerosis by mediating cholesterol efflux (21). Additionally, impaired HDL-C function can hinder Aβ clearance (22). Compared to moderate TC levels, both long-term low and long-term high TC levels are associated with an increased risk of developing cognitive impairment within 6 years (23). Cholesterol serves as an essential component of neuronal cell membranes and synaptic structures, participating in neurotrophic factor signaling and lipid raft formation; consequently, moderate cholesterol levels may exert neuroprotective effects by sustaining synaptic activity and signaling (24). While peripheral cholesterol itself cannot cross the blood–brain barrier (BBB), BBB dysfunction associated with aging can permit its entry into the brain, thereby influencing cholesterol homeostasis (25). Furthermore, elevated TC levels correlate with increased levels of metabolites like 24S-hydroxycholesterol and 27S-hydroxycholesterol, both of which can damage the BBB. This compromise allows inflammatory factors and serum cholesterol to infiltrate the brain (26). Studies demonstrate that lipid-lowering drugs can mitigate the rate of cognitive decline in patients with cognitive impairment (27), potentially achieved through modulating β-secretase and *γ*-secretase activity and reducing neuronal levels of Aβ42 and Aβ40 peptides (28). HDL-C, primarily synthesized in the liver, is crucial for reverse cholesterol transport (RCT), facilitating the return of circulating cholesterol to the liver for biliary excretion, thereby reducing the risk of cholesterol accumulation and vascular damage. Cholesteryl ester transfer protein (CETP), a key component of HDL-C, is central to RCT (29). Liu et al. (30) demonstrated significantly lower serum HDL-C levels in patients with vascular dementia (VD) compared to healthy controls and those with Alzheimer’s disease. However, when HDL-C exceeds 2 mmol/L, the risk of cognitive impairment significantly increases (31). Small HDL particles within the cerebrospinal fluid (CSF) positively correlate with cognitive function (32), as they facilitate lipid exchange between plasma and CSF, promote neuronal membrane lipid remodeling, enhance synaptic plasticity, and accelerate amyloid-β clearance (33). Conversely, excessively high plasma HDL-C levels promote an increase in HDL particle size (34), disrupting the homeostasis between plasma HDL and CSF. This disruption interferes with the neuroprotective functions of small HDL particles and ultimately contributes to cognitive impairment.

Recent studies indicate that patients with familial hypercholesterolemia (FH) exposed to lifelong elevated LDL-C levels develop subclinical vascular damage by increasing pulse wave velocity and intima-media thickness, ultimately contributing to cognitive impairment (35). Non-high-density lipoprotein cholesterol (non-HDL-C) was initially established as a predictor of cardiovascular disease risk. Its predictive value for all-cause mortality and cardiovascular mortality has been demonstrated to be significantly superior to that of LDL-C (36). Recent research evidence supports a close association between serum non-HDL-C levels and cerebrovascular disease. Studies have demonstrated that non-HDL-C levels are significantly higher in individuals with cognitive impairment compared to cognitively normal groups and control groups, and non-HDL-C levels show a negative correlation with Montreal Cognitive Assessment (MoCA) scores (37). Elevated non-HDL-C levels constitute an independent risk factor for the development of cognitive impairment following acute ischemic stroke (38). Increased levels of non-HDL-C and LDL-C within the body promote the development of atherosclerosis, subsequently triggering arterial lumen narrowing, increased plaque formation, and hemodynamic abnormalities. This vascular pathology can lead to impaired blood supply in the thalamic region, potentially damaging the structure and function of the hippocampus and adjacent brain areas, ultimately resulting in cognitive dysfunction. Therefore, the core mechanism underlying cognitive dysfunction induced by abnormally elevated non-HDL-C levels is likely closely linked to this atherosclerotic process (39). In recent years, increasing attention has been focused on innovative lipid-lowering therapies and their role in reducing LDL-C in high cardiovascular risk populations, such as those with FH. The first study exploring the effects of inclisiran on both lipid profiles and pulse wave velocity in FH subjects demonstrated that non-HDL cholesterol as well as PWV could be improved using statins, ezetimibe or PCSK9 inhibitors. Early treatment with lipid-lowering therapies ameliorates lipid profiles and vascular health, and could also reduce the onset of subclinical vascular damage (40).

The NHHR precisely captures the balance between the atherogenic non-HDL-C and atheroprotective HDL-C, providing a more comprehensive assessment of metabolic status. Although total cholesterol did not differ significantly between groups, NHHR levels were markedly elevated in the cognitive impairment group (*p* < 0.001). This evidence demonstrates that NHHR is superior to individual lipid markers in predicting atherosclerosis-related diseases.

Additionally, our study found that uric acid was a protective factor for cognitive function (OR = 0.99, 95% CI: 0.99–0.99, *p* = 0.002). One of the key physiological functions of serum uric acid (SUA) is its role as an endogenous antioxidant, neutralizing various reactive oxygen and nitrogen species to exert a neuroprotective effect against oxidative damage (41). SUA has also been found to mitigate the toxic effects associated with the aggregation of Aβ and tau proteins (42). While substantial evidence supports an association between elevated serum uric acid (SUA) levels and improvements in various cognitive functions, particularly in memory, language, and numerical cognition (43), it is crucial to recognize the dual nature of SUA’s impact on cognitive health. Research conducted in populations with type 2 diabetes has revealed a U-shaped curve relationship between SUA and the risk of developing mild cognitive impairment (MCI), with an inflection point at 388.63 μmol/L. Below this threshold, SUA exhibits a protective effect; above it, SUA transforms into a risk factor (44). Other studies also caution that elevated SUA may generally increase the incidence of vascular or mixed dementia in the elderly population, indicating that comorbid conditions profoundly influence the actual role of SUA (45). Consequently, to provide optimal protection for cognitive function, efforts must be directed toward finding an optimal window: leveraging the potential benefits of SUA within the normal range while remaining vigilant about the possibility that hyperuricemia may indirectly increase dementia risk by triggering other diseases.

The therapeutic potential of NHHR warrants exploration. Lipid modulation targeting NHHR may represent a novel strategy for improving cognitive outcomes. However, this field requires further clinical trials and research to validate its efficacy and safety, and to establish optimal therapeutic approaches. Future investigations may offer promising avenues for cognitive impairment management. Despite these novel findings on the NHHR-cognitive impairment association, several limitations warrant consideration: First, cognitive assessment was conducted only using the MMSE, which is less sensitive than the MoCA for detecting vascular cognitive impairment, particularly impairments in executive function. Additionally, the retrospective design precludes establishing causal relationships between variables, necessitating confirmation through prospective studies. Moreover, the modest sample size may introduce selection bias. Future prospective investigations will explore causal links between these variables.

## Data Availability

The original contributions presented in the study are included in the article/supplementary material, further inquiries can be directed to the corresponding author.
